# Regional Variations in the Intra- and Intervertebral Trabecular Microarchitecture of the Osteoporotic Axial Skeleton with Reference to the Direction of Puncture

**DOI:** 10.3390/diagnostics14050498

**Published:** 2024-02-26

**Authors:** Guido Schröder, Thomas Mittlmeier, Patrick Gahr, Sahra Ulusoy, Laura Hiepe, Marko Schulze, Andreas Götz, Reimer Andresen, Hans-Christof Schober

**Affiliations:** 1 Department of Traumatology, Hand and Reconstructive Surgery, Rostock University Medical Center, Schillingallee 35, 18057 Rostock, Germany; 2 Faculty of Medicine, University of Rostock, Ernst-Heydemann-Str. 8, 18057 Rostock, Germany; 3 Institute of Anatomy, Rostock University Medical Center, Gertrudenstraße 9, 18057 Rostock, Germany; 4 Institute of Anatomy and Cell Biology, University of Bielefeld, Morgenbreede 1, 33615 Bielefeld, Germany; 5 Institute for Biomedical Engineering, University Medical Center Rostock, Friedrich-Barnewitz-Straße 4, 18119 Rostock-Warnemuende, Germany; 6 Institute for Diagnostic and Interventional Radiology/Neuroradiology, Westkuestenklinikum Heide, Academic Teaching Hospital of the Universities of Kiel, Luebeck und Hamburg, Esmarchstraße 50, 25746 Heide, Germany; 7 OrthoCoast, Practice for Orthopedics and Osteology, Hufelandstraße 1, 17438 Wolgast, Germany

**Keywords:** micro-CT, osteoporosis, insufficiency fracture, cancellous bone structure, spine

## Abstract

Background: Trabeculae in vertebral bodies are unequally distributed within the cervical spine (CS), the thoracic spine (TS), and lumbar spine (LS). Such structures are also unequally distributed within the individual vertebrae. Exact knowledge of the microstructure of these entities could impact our understanding and treatment of fractures caused by osteoporosis and possibly improve surgical approaches. Appropriate investigations could help clarify the pathomechanisms of different forms of osteoporotic vertebral fractures, as well as different changes in morphological findings like the trabecular bone score (TBS). In the present study, we applied punctures to the craniocaudal and ventrocaudal directions and obtained cylinders of cancellous bone from the central portions and marginal regions of cervical vertebrae 5 and 6, thoracic vertebrae 8 and 12, and lumbar vertebrae 1 and 3. We systematically analyzed these samples to determine the bone volume fraction, trabecular thickness, separation, connectivity density, degree of anisotropy, and structure model index. Methods: Using an 8-gauge Jamshidi needle, we obtained samples from three quadrants (Q I: right margin; Q II: central; Q III: left margin) in the frontal and transverse plane and prepared these samples with a moist cloth in a 1.5 mL Eppendorf reaction vessel. The investigations were performed on a micro-CT device (SKYSCAN 1172, RJL Micro & Analytic Company, Karlsdorf-Neuthard, Germany). All collected data were analyzed using the statistical software package SPSS (version 24.0, IBM Corp., Armonk, NY, USA). Student’s *t* test, the Wilcoxon–Mann–Whitney test, the Chi-squared test, and univariate analysis were used for between-group comparisons. The selection of the test depended on the number of investigated groups and the result of the Shapiro–Wilk test of normal distribution. In the case of statistically significant results, a post hoc LSD test was performed. Results: In total, we obtained 360 bone samples from 20 body donors. The craniocaudal puncture yielded data of similar magnitudes for all investigated parameters in all three quadrants, with the highest values observed in the CS. Comparisons of the ventrodorsal and craniocaudal microstructure revealed a significantly lower trabecular density and a significantly higher degree of anisotropy in the craniocaudal direction. Conclusions: The results presented different distributions and behaviors of trabecular density, with lower density in the mid-vertebral region over the entire breadth of the vertebrae. Reduced trabecular density caused a higher degree of anisotropy and was, therefore, associated with a lower capacity to sustain biomechanical loads. Fractures in fish vertebrae were easily explained by this phenomenon. The different changes in these structures could be responsible, in part, for the changes in the TBS determined using dual-energy X-ray absorptiometry. These results confirm the clinical relevance of the TBS.

## 1. Introduction

Osteoporosis (OP) is a metabolic disease of the skeletal system, marked by reduced bone mass and a poor microarchitecture of bone [1]. The frequency of clinically relevant osteoporotic vertebral fractures (VFs) is about 1.4 million cases per year [2], and the consequences of these fractures for patients are significant. Osteoporotic VFs can cause pain, kyphotic deformations, poor quality of life, loss of independence, referral to a care home, and higher morbidity and mortality rates [3,4]. The thoracic vertebrae (TV) 7, 8, and 12 and the lumbar vertebra (LV) 1 are especially affected by vertebral fractures [5]. Osteoporotic fractures of vertebrae above TV 4, especially in the cervical spine (CS), have only been reported in rare cases [6,7].

The present investigation is focused on determining the reasons for structural changes in various regions of the vertebrae and their potential effects on deformities and fractures in advanced age. The trabecular portions of vertebrae decrease markedly with advancing age, which is largely responsible for VFs. However, the etiology of VFs remains ambiguous, despite the high prevalence of such fractures [4]. The trabecular structures of vertebral bodies are distributed unequally in the CS, thoracic spine (TS), and lumbar spine (LS), as well as within the individual vertebra [8]. Additionally, men and women differ in terms of their vertebral microarchitecture [8,9]. With advancing age, the trabecular structure undergoes morphological changes [9,10]. The biomechanical competence of the vertebrae is produced by the geometry of cortical bone and the density, thickness, and arrangement of the trabeculae. According to current knowledge, we have limited information on the density and arrangement of trabeculae in all portions of the spine [8,11]. Few investigations have addressed the question as to whether intra- or intervertebral differences in the thickness, density, number, and separation of trabeculae in the CS compared to those in the TS and LS are responsible for their corresponding susceptibility to fractures in the presence of osteoporosis. To the best of our knowledge, no investigation has provided a comparative description of the various puncture directions in the frontal and transverse plane or the individual regions of the vertebrae. A precise microstructural analysis of these factors could influence not only the understanding, diagnosis, and treatment of osteoporotic VFs but also surgical procedures. Furthermore, the present study provides hitherto unknown details about the pathomechanisms of various types of VFs, such as fish-shaped, wedge-shaped, and flat vertebrae. We investigated and compared 120 vertebrae to determine the bone volume fraction of their trabeculae, their thickness, their separation, their connectivity density, their degree of anisotropy, and their structure model indexes using micro-computed tomography (micro-CT). We also determined bone mineral density by performing quantitative computed tomography (QCT) investigations of the LS.

## 2. Materials and Methods

### 2.1. Design and Group Allocation

We conducted a multicenter clinical investigation of an intervention group. The assignment to groups depended on the location of the vertebrae in the individual segments of the spine.

### 2.2. Recruitment and Ethics Approval

Participants of the study were people who had joined the body donor program at the Medical University during their lifetimes and had voluntarily consented to their bodies being used for scientific research after their demise. We had limited medical information about the donors. The principal known fact was the cause of death. The methods used to obtain human tissue were carried out in accordance with the ethical standards of the Declaration of Helsinki. This study was reviewed and approved by the regional ethics committee for medical research (approval no. A 2017-0072).

### 2.3. Inclusion and Exclusion Criteria

The clinical investigation included body donors from whom we extracted CV 5, CV 6, TV 8, TV 12, LV 1, and LV 3. The vertebrae were free from anatomical deformities or severe bone disease, such as tumors or bone metastases. Furthermore, the body donors were expected to be of advanced age (older than 65 years of age), and their spines were required to have no signs of growth retardation, Paget’s disease, vertebral fusions, or block vertebrae. Likewise, body donors who had undergone surgery on the spine with foreign material were excluded from the investigation.

### 2.4. Extraction of Spines and Cancellous Bone

**Figure 1:**
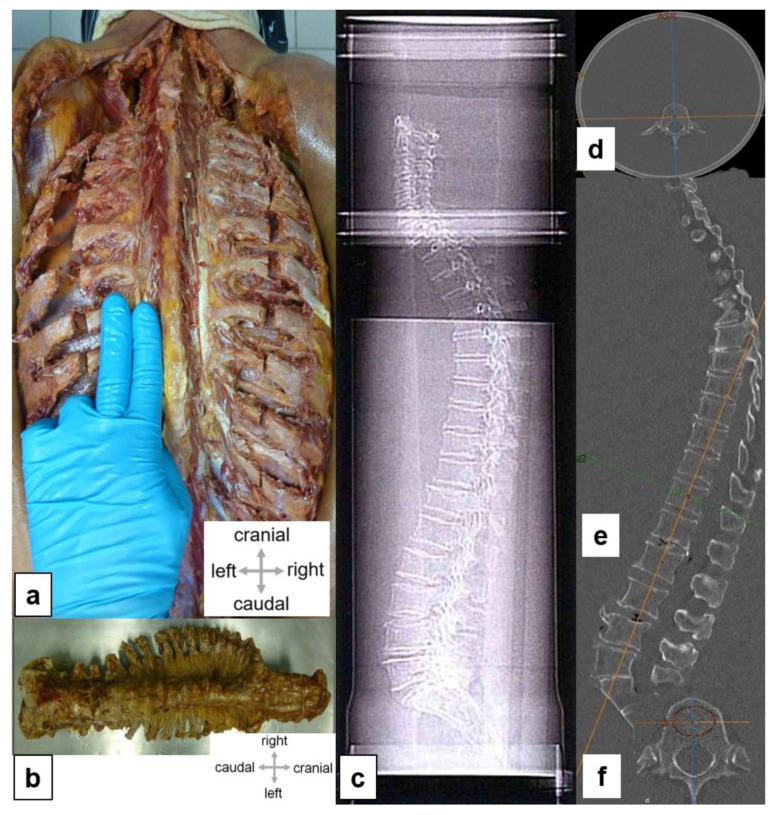
Experimental setup. (**a**) Skin incision along the spinous process from the sacrum to the occiput (superior nuchal line); skin and subcutaneous fatty tissues were shifted laterally. The subsequent lateral tract of the autochthonous spinal muscles was removed, and the ribs were exposed. The spinal muscles have been largely removed; the intercostal spaces have been emptied in the paravertebral aspect. The cervical spine has been mobilized in the retropharyngeal space, and the scalene muscles have been transected. Transection of all ribs about 1.5 inches wide in the paravertebral aspect of the spinous processes, along the scapular line. Separation of the parietal pleura from the inside of the ribs and wedge-shaped incision in the sacrum. (**b**) Complete specimen of the spine. (**c**) Position of an embedded spine in a PVC water phantom. For simulations of the soft tissue mantle, the spine was embedded, as far as possible in non-aerated conditions, in water. (**d**) The transverse diameter of the phantom was 25 cm. (**e**) Lateral scanogram; To detect fractures, we obtained a sagittal reconstruction that was oriented as far as possible plane-parallel to the base and cover plates. (**f**) Image reformation and measurement of bone mineral density in a axial CT section.

Depending on the postmortal stage, the left femoral arteries of the cadavers were perfused with a 96% ethanol solution at a pressure of 0.5 bar. The spines were then conserved in a free-floating state in an aqueous 0.5% phenol solution. The body donors were placed in prone position, and the spines were extracted (see Figure 1a,b). The extracted samples were stored in a 70% ethanol solution at 4 degrees centigrade to perform subsequent imaging studies and cancellous bone punctures. Cancellous bone was punctured from the ventrodorsal (VD) and craniocaudal (CC) aspects in the center and the marginal regions of the vertebrae. Precise positioning was confirmed using CT to account for any existing fusion fractures. In the identified fusion fractures, we found sufficient non-compressed cancellous tissue that was used to extract biopsy specimens. Using a Jamshidi needle^®^ (8 gauge, 3.263 mm), we obtained a total of 360 cancellous bone cylinders from the ventromedial, craniomedial, and marginal areas from 120 previously prepared vertebrae. These samples were placed in a 1.5 mL Eppendorf reaction vessel for further investigation.

### 2.5. Diagnostic Imaging

#### 2.5.1. CT and QCT

To create realistic conditions for clinically precise anthropomorphic measurements, the extracted spines (see Figure 1a,b) were embedded carefully in a plexiglass water phantom measuring 25 cm in diameter and 125 cm in length (see Figure 1c,d). Care was taken to avoid air pockets.

All donor spines were subjected to a high-resolution spiral CT investigation (GE Revolution EVO/64-slice CT/lateral scanogram, Solingen, Germany) with an axial slice thickness of less than 1 mm and axial and sagittal reformations with slice thicknesses of 2 mm. Vertebral deformities were identified and graded based on the sagittal reformations (see Figure 1e) [12]. Bone mineral density (BMD) was determined via quantitative CT (GE Revolution EVO/64-slice computed tomography device and Mindways software (version 4.2.3), 3D Volumetric QCT Spine, Austin, TX, USA) (see Figure 1f). The bone mineral density of cancellous bone was determined using a volume block at the levels of LV 1, LV 2, and LV 3. The mean value, given in mg/cm^3^, was used to determine the presence of osteoporosis.

#### 2.5.2. Micro-CT Images and Evaluation of Microarchitecture

The cancellous bone cylinders were examined on a micro-CT device (SKYSCAN 1172, RJL Micro & Analytic GmbH, Karlsdorf Neuthart, Germany). For this analysis, we performed a flat-field correction and compared the images with phantoms (reference images) with densities of 0.25 g/cm^3^ and 0.75 g/cm^3^. The settings for the scanning process were established as follows: filter: AI 0.5; resolution: 640 × 512 pixels; pixel size: 19.9 µm; isotropic nominal voxel size: 35 mm (field of view: 70 mm; X-ray source: 100 kV and 100 µA).

**Figure 2:**
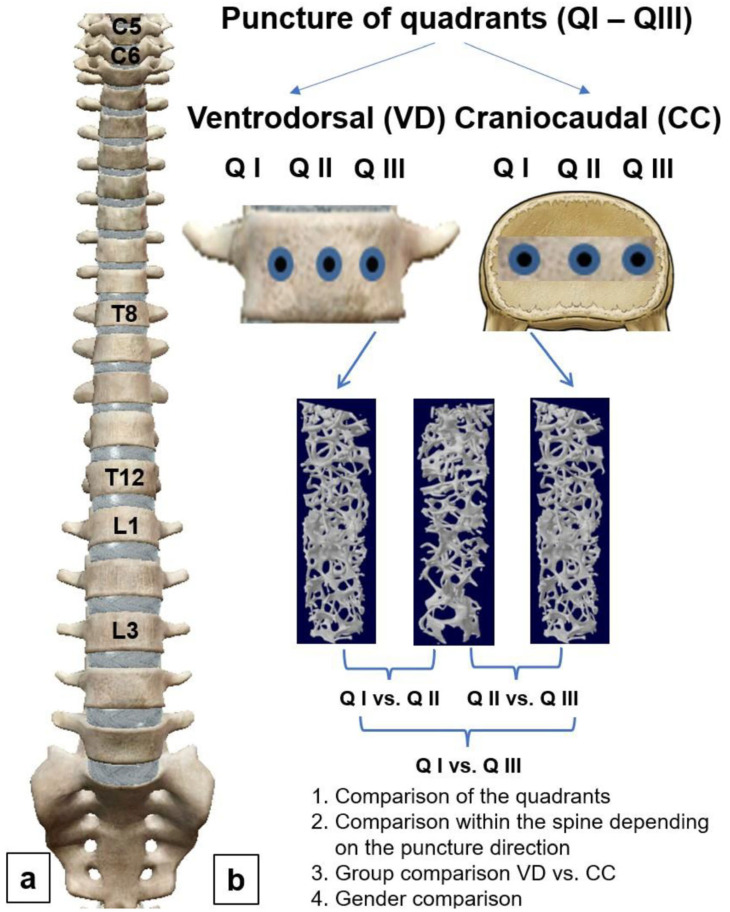
Flow diagram of method. (**a**) Longitudinal comparisons of individual segments of the spine for each quadrant; (**b**) cross-comparisons within the individual segments of the spine; Q I: Quadrant I; Q II: Quadrant II; Q III: Quadrant III. Statistical comparisons were made between quadrants (cross-comparison) and between the individual segments of the spine (longitudinal comparison). In addition, group comparisons were performed between genders and the different directions of puncture.

The trabecular region of interest was defined manually to exclude the cortical components of the vertebral bodies. The following parameters of trabecular microarchitecture were measured: bone volume fraction (BVF, %), trabecular thickness (Tb.Th, µm), trabecular separation (Tb.Sp, µm), connectivity density (Conn.D, mm^−3^), degree of anisotropy (DA, 0 = isotropic; 1 = anisotropic), and structure model index (SMI, 0 = plate structure; 3 = rod structure). Figure 2a provides an overview of the analyzed cervical (CV), thoracic (TV), and lumbar vertebrae (LV). Figure 2b shows the methodical procedure used for the micro-CT examination.

### 2.6. Statistics

All data were analyzed with the statistical software package SPSS (Version 24.0, IBM Corp., Armonk, NY, USA). We used Student’s *t*-test, Wilcoxon–Mann–Whitney U test, chi-squared test, and unifactorial analysis of variance for comparisons between groups. The selection of the test depended on the number of groups being investigated and the result of the Shapiro–Wilk test of normal distribution. In the case of statistically significant results, we initiated a post hoc LSD test. Quantitative characteristics are displayed as the means (M), standard deviation (SD), and number (*n*) of available observations. Furthermore, depending on the scale level, we performed Pearson correlation analyses and determined the eta coefficient. *p*-values were obtained from statistical two-sided tests and generally considered significant when they were less than 0.05. Furthermore, a Bland–Altman plot was charted as a graphic comparison of the two types of punctures (VD vs. CC) used for BVF. The mean of two measurements was used to establish differences.

## 3. Results

In total, we extracted 20 spines from human body donors. These donors consisted of six men and fourteen women aged 66 to 91 years (mean age, 79.4 ± 6.4 years). The available medical histories were limited to the cause of death. A short summary of medical histories is provided in Table 1 (see Table 1).

### 3.1. QCT

**Figure 3:**
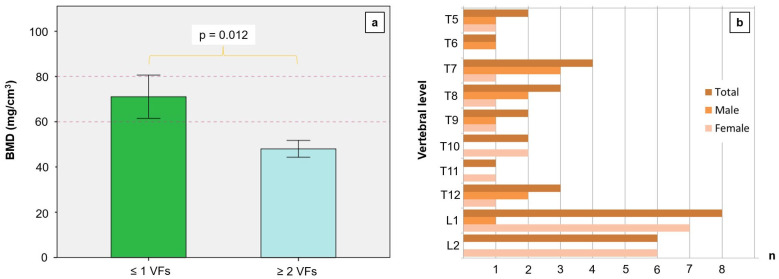
Mean lumbar bone mineral density (BMD in mg/cm^3^) with reference to the number of vertebral fractures (VFs). A BMD below 80 mg/cm^3^ is defined as osteoporosis; the risk of fractures is significantly higher at values below 60 mg/cm^3^. A markedly reduced BMD leads to more numerous fractures in the thoracic region, thoracolumbar junction, and lumbar region. (**a**) We found no fractures in the cervical segments of the investigated spines. (**b**) Fracture numbers depending on gender and the location of vertebrae. We found no fractures above thoracic vertebra 5 and below lumbar vertebra 2.

The mean bone mineral density of the 20 spines, measured on lumbar vertebrae 1 to 3, was 57.2 ± 22.4 mg/cm^3^, which indicates severe osteoporosis. At a bone mineral density below 60 mg/cm^3^, there were significantly more fusion fractures in the thoracic and thoracolumbar regions (see Figure 3a,b). We found no fractures in the CS.

The correlation analysis between the age of the body donors and BMD yielded a negative coefficient and a statistical trend (r = −0.423, *p* = 0.063). On the other hand, the number of fractures was significantly correlated with BMD (r = −0.520, *p* = 0.019). Likewise, the eta coefficient was significant when examining the correlation between the probability of a vertebral fracture and BMD (ɳ = 0.522, *p* = 0.018).

### 3.2. Micro-CT

In the VD group, the central quadrant (Q II) had a significantly lower BVF than that of marginal quadrants Q I and Q III. A group comparison of BVF yielded a significant difference between Q I and Q II (*p* = 0.016) and a statistical trend between Q II and Q III (*p* = 0.095). A comparison of Q I and Q III, on the other hand, yielded no significant difference (*p* > 0.05). In the CC group, BVF did not differ significantly between the quadrants (see Table 2).

A group comparison of Tb.Th showed no significant difference between quadrants in the CC and VD groups. The trabeculae also had similar thickness values (see Table 2).

The trabeculae from Q I to Q III were at a similar distance to each other, independent of the direction of the puncture (see Table 2).

The density of the trabecular network was similar in all quadrants in the CC and VD groups (*p* > 0.05).

The degree of anisotropy was lowest in the central quadrants in the VD group (see Table 2). Upon a comparison of the groups, a significant difference was observed between Q 1 and Q II, as well as between Q III and Q II (*p* < 0.001). However, we found no significant difference between the marginal regions (*p* > 0.05). A group comparison of the CS revealed a significant difference between Q I und Q II (*p* < 0.001) and between Q III and Q II (*p* < 0.001). Furthermore, a comparison of the marginal regions Q I and Q III yielded a significant difference (*p* = 0.047). The differences in the arrangement of cancellous bone in the TS were significant between Q I and Q II and between Q III and Q II (*p* = 0.001). However, a comparison of values in the marginal areas revealed no significant difference (*p* > 0.05). A group comparison of DA in the LS showed a significant difference between Q I and Q II (*p* = 0.001) and between Q III and Q II (*p* = 0.001). The difference between Q I and Q III, on the other hand, was not significant (*p* > 0.05). For the CC group, we registered no significant difference between quadrants based on the degree of anisotropy.

Analyses of the SMI values yielded no significant differences between the quadrants of the CC group and VD group (*p* > 0.05). These data are summarized in Table 2.

In general, we observed a positive significant correlation between BVF and Tb.Th (r = 0.783, *p* < 0.001). BVF was significantly negatively correlated with DA (r = −0.483, *p* = 0.031). DA, in turn, was significantly negatively correlated with Tb.Th (r = −0.474, *p* = 0.035). Furthermore, a significant negative correlation was observed between Tb.Sp and Conn.D (r = −0.642, *p* = 0.002).

### 3.3. Subgroup Analysis

Using a subgroup analysis, we performed longitudinal comparisons of trabecular parameters in the various segments of the spine for each group. Differences in the architecture of cancellous bone with reference to the location of the vertebrae are summarized in Table 3.

In the CS, we found more numerous trabeculae per volume only in the CC group. A pairwise comparison of BVF showed significant differences between the CS and TS and between the CS and LS (*p* < 0.001). The comparison of the TS and LS, on the other hand, yielded no significant difference (*p* > 0.05). Furthermore, no significant difference in BVF was observed between the individual segments of the spine in the VD group (see Table 3).

A group comparison of Tb.Th revealed significantly thicker trabeculae in the cervical vertebrae compared to the thoracic (*p* = 0.003) and lumbar vertebrae, albeit only in the CC group (*p* = 0.049). In the VD group, we found no significant differences between the various segments of the spine (*p* > 0.05).

A longitudinal comparison of the segments of the spine showed Tb.Sp to be similar in the CC and VD groups (*p* > 0.05, see Table 3).

The density of the trabecular network in the CS was greater than that in the TS (*p* = 0.001) and LS (*p* = 0.001), albeit only in the CC group. In contrast, the difference between the TS and LS was not significant (*p* > 0.05). The density of the trabecular network in the VD group did not differ between the various segments of the spine (*p* > 0.05).

The trabecular degree of anisotropy in the CS was greater than that in the TS (*p* = 0.001) and LS (*p* = 0.007) but only in the CC group. In contrast, the difference between the TS and LS was not significant (*p* > 0.05). Group comparisons of the degree of anisotropy in the VD group revealed no significant difference (*p* > 0.05, see Table 3).

In the CC group, a group comparison of SMI showed significantly more rod-shaped trabeculae in the cervical vertebrae compared to thoracic (*p* < 0.001) and lumbar vertebrae (*p* < 0.001). In the VD group, we also observed significant differences between the various segments of the spine. Cervical vertebrae had significantly more rod-shaped trabeculae than thoracic (*p* = 0.007) or lumbar vertebrae (*p* < 0.001). In all of the punctured quadrants, the micro-CT parameters yielded significant differences between the CC and VD groups in terms of BVF (*p* = 0.018), Tb.Th (*p* < 0.001), and DA (*p* < 0.001). A group comparison is shown in Table 4.

**Figure 4:**
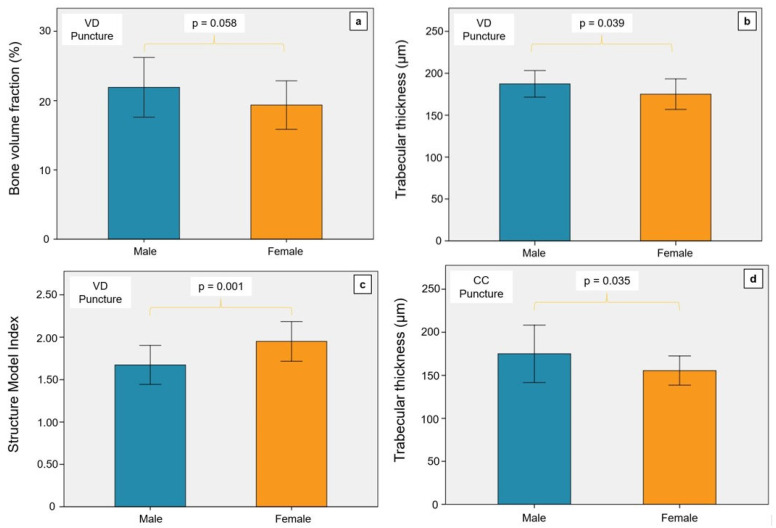

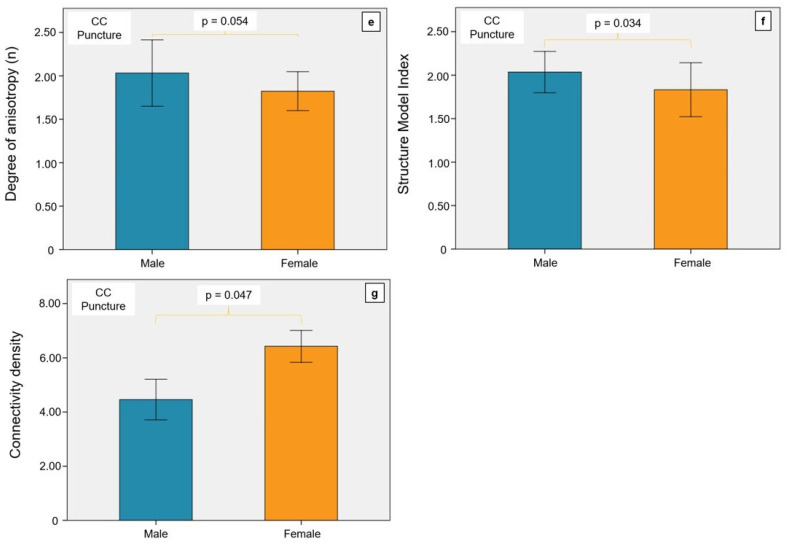
Illustration of the gender comparison for the relevant parameters of the micro-CT examination. VD, ventrodorsal puncture; CC, craniocaudal puncture. (**a**) The BVF was not significantly different in the VD puncture in the men and women examined. There was a statistical trend with a tendency towards higher BVF in men. (**b**) The trabeculae of the men were significantly thicker in the VD puncture than in the women. (**c**) The Structure Model Index of the women was significantly higher in the VD puncture than in the men. (**d**) In the CC puncture, men also had significantly thicker trabeculae than women. (**e**) The degree of anisotropy did not differ between men and women in the CC puncture. (**f**) The Structure Model Index of men was significantly higher than that of women in the CC puncture. (**g**) The connectivity density was significantly higher in women than in men in the CC puncture.

The subgroup analysis was also used to evaluate gender-specific differences with reference to the direction of the puncture. The BVF of men in the VD group was significantly higher than that of women (see Figure 4a). This result could be interpreted as a statistical trend (*p* = 0.058). Male body donors had significantly thicker trabeculae, independent of the direction of the puncture (CC group, *p* = 0.035; VD group, *p* = 0.039; see Figure 4b,d).

In the VD group, a significantly lower SMI was observed in men (*p* = 0.001) than in women (see Figure 4c). On the other hand, the SMI of men in the CC group was significantly higher than that of women (*p* = 0.034, see Figure 4f). The DA of men was higher than that of women in the CC group (see Figure 4e). This outcome was not significant but may be interpreted as a statistical trend (*p* = 0.054). However, the connectivity density of women in the CC group was significantly higher than that of men (*p* = 0.047, see Figure 4g).

**Figure 5:**
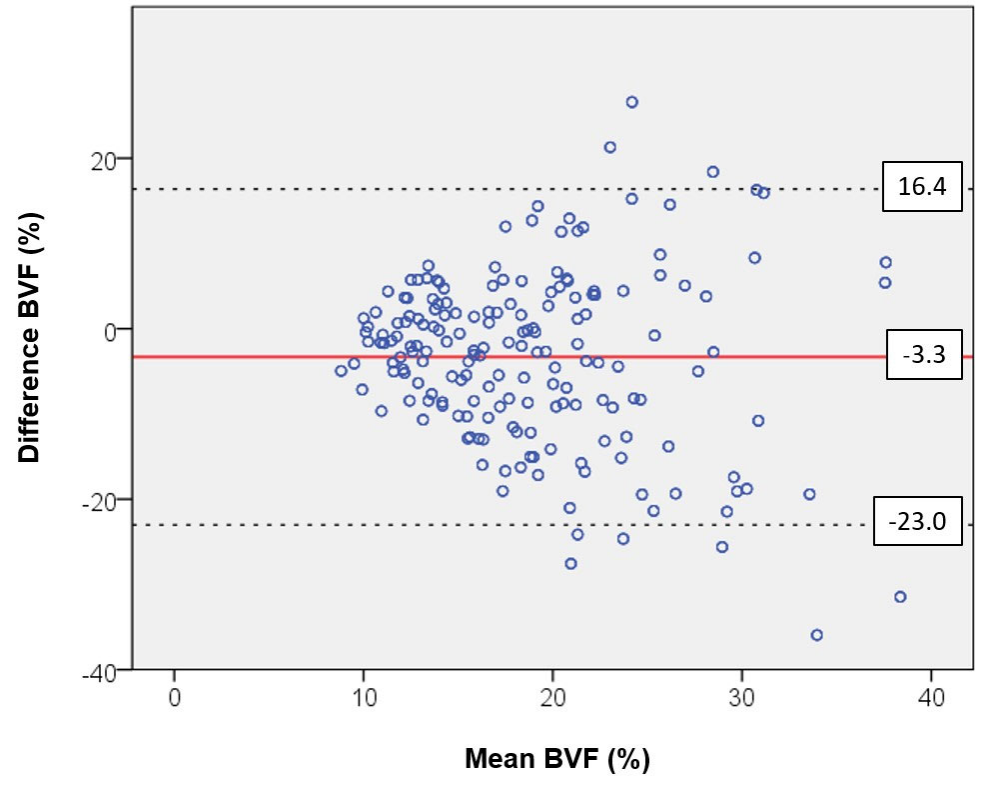
Bland–Altman plot showing the differences between the ventrodorsal and craniocaudal measurements for the bone volume fraction (BVF) as a function of the mean value. The bias of −3.3 is at a low level. The scatter analysis shows no random scattering of the data around the mean value. Instead, there is a professional bias in the form of a funneling of the data, which produces comparable values at a low BVF but strongly deviating values at a higher BVF. The 95% confidence interval indicates the limits of agreement. It is decisive for the fluctuation range of the differences.

The Bland–Altman plot shows the differences between ventrodorsal and craniocaudal measurements of BVF with reference to the mean value. The bias is low at −3.3. The analysis of variance revealed no incidental variation in data around the mean value. Instead, we registered a funnel-shaped professional bias. At a low BVF, we observed similar values but at a higher BVF with markedly deviating values. The 95% confidence interval shows the limits of concordance, which is critical for determining the variability of differences (see Figure 5).

## 4. Discussion

The present investigation enabled a comparison of trabecular bone from different intravertebral regions and from all segments of the spine. Samples for analysis were extracted from 20 body donors aged 66 to 91 years. To the best of our knowledge, this is the first investigation to provide data from different puncture directions in the frontal and transverse plane. The BMI in the overall group was normal. A BMI below 22 is associated with an increasing fracture rate [13]. All investigated probands had a bone mineral density below 80 mg/cm^3^ measured using bone densitometry, which was considered a sign of osteoporosis in all subjects. Values below 60 mg/cm^3^ were always associated with fusion fractures. A major factor that contributed to these findings was the age of the donors, as a higher fracture rate is observed among those beyond the age of 70 years [14]. The fractures were typically distributed in the thoracolumbar and lumbar portions of the spine.

A notable aspect of our data was the higher BVF in the marginal regions of the spine, which was primarily observed in the ventrodorsal puncture group (VD group). In contrast, the puncture in the craniocaudal direction (CC group) showed that the BVF in the marginal areas was similar in various quadrants. These data provide a potential explanation for fractures in fish vertebrae.

These data may also explain the findings of different gray-scale parameters of bone texture using a modified experimental variogram that can be extracted from dual-energy X-ray absorptiometry (DXA) images, even under normal bone mineral density.

In a recent investigation, Schröder et al. [15] studied cancellous bone cylinders from 3 quadrants of 11 body donors aged 79.1 years on average. The authors performed punctures on 242 vertebrae in the ventrodorsal direction and extracted 726 cancellous bone cylinders. The authors observed significantly higher bone density in the trabecular structure of the marginal regions of the cervical spine (CS) than that in the thoracic (TS) and lumbar spine (LS). Grote et al. [11] showed that the loss of bone volume with advancing age is lowest in the CS. In cervical vertebrae 3 and 4, the authors registered no significant age-related loss of trabecular density. In a histologic study of osteocyte density in the trabecular bone of all vertebral segments, Schröder et al. [16] found no difference in the osteocyte number based on age or gender.

The location of sampling is significant for the allocation of empirical results. Chen et al. [9] and Gong et al. [10] found a lower BVF in the central and anterosuperior portions of the vertebrae compared to that in the posterior region. Banse et al. [17] also assumed that the region with the lowest density is primarily located in the upper and anterior half of the vertebra. Considering these observations, we extracted our samples from the center of, front of, and above the vertebrae. We assumed that the structures with the lowest density would be covered by this approach. Knowledge of regional differences in microstructural properties is relevant for the assessment of age- and gender-related bone loss in vertebrae. Structural associations will improve our understanding of various fracture forms and risks [18]. Microdamage in trabeculae, caused partly by a reduction in BVF, was found to play a role [19]. BVF was determined from Tb.N and Tb.Sp. A reduction in Tb.N due to age causes an increase in Tb.Sp. [9]. In the present investigation, the CC group had thicker trabeculae in the CS compared to the results in the TS and LS. Men had a higher Tb.Th than women, independent of the direction of the puncture.

In general, the thickness of vertical struts in trabecular vertebral bone was 100–200 µm, whereas the Tb.Sp of vertical trabeculae was as high as 600–900 µm [17,20,21]. Our own values are more or less consistent with the data reported in the published literature. For the analysis of the trabecular bone structure, however, one should differentiate between vertical and horizontal trabecular components. Loads exerted on the vertebrae are mainly borne by vertical trabeculae, whereas horizontal trabeculae serve to prevent vertical trabeculae from turning over [22]. This perspective was supported by fine element analyses of human vertebral bone samples, which showed that vertical trabeculae are exposed to much greater stresses than horizontal trabeculae in the presence of normal loads [20,23]. Consequently, trabecular thickness and bone volume fractions must be quantified separately for horizontal and vertical trabeculae [1].

The Conn.D in the CC group was significantly higher in all quadrants of the CS than that in the TS or LS. This finding agrees with previous studies [8,24]. With the aid of a finite element model, Kinney und Ladd [25] investigated the association between connectivity density and the elasticity module of trabecular bone. The authors reported a linear association between loss of the elasticity module and an overall loss of connectivity. These data indicate that the restoration of mechanical function is dependent on the preservation or restoration of trabecular connectivity [25]. In the present investigation, Conn.D was negatively correlated with Tb.Sp. In other words, connectivity density increased when the distance between trabeculae was reduced. The outcome of the gender comparison after craniocaudal puncture remains difficult to explain. Women had a higher Conn.D than men. The body weights of the women in this sample were possibly a causal factor.

The DA in the VD group was significantly lower in the central quadrant than that in the marginal regions of the spine, independent of the spinal segment being investigated.

These data agree with those reported by Chen et al. [9]. In the intravertebral aspect, the authors found no significant differences in DA. For VD puncture, Schröder et al. [15] also reported lower DA values in the central quadrant compared to those in the marginal areas. The higher DA of men compared to women in the CC group agrees with the lower connectivity registered in men.

In the present investigation, the quadrant comparison in the CC group yielded no significant differences, whereas the longitudinal comparison revealed significantly lower DA values in the CS. An anisotropic structure is caused by the deterioration of trabecular vertebral architecture, which, in turn, indicates greater susceptibility to fractures [20].

For the SMI, a comparison of quadrants in the CC group and VD group yielded similar values. On the other hand, the longitudinal comparison revealed significant differences in the CC group and VD group. Lower SMI values were observed in the CS compared to those in the TS and LS. Chen et al. [9] also registered no significant difference in the intravertebral aspect. However, the authors did not perform a longitudinal comparison of the various segments of the spine. SMI was introduced by Hildebrand and Rüegsegger [26] and quantifies the shape of the trabecular structure. As the microarchitecture of bone includes plate-shaped and rod-shaped structures, the SMI is intended to demonstrate a tendency towards one form or the other [26]. An SMI of 0 indicates that the plates have an ideal structure, whereas a value of three indicates that the rods have an ideal structure. Accordingly, independent of the direction of puncture, we found more plate-shaped structures in the CS, which contributed to the stability of this spinal segment. However, the lower SMI of women in the CC group remains inexplicable.

The load-bearing capacity of a segment of the spine depends on the material properties of the vertebrae and their geometric dimensions. As the material properties of bone tissue are predetermined in the widest sense of the term, the dimensions of a vertebra may be viewed as a result of adjustments to internal and external forces acting on the vertebra. The load-bearing capacity of vertebrae increases with their size [24]. Indeed, the mere location of lumbar vertebrae causes them to be loaded to a greater extent because the effective physical load of the body acting on the vertebrae increases towards the caudal aspect [27].

However, the spine is not a rigid structure and is not subjected to dynamic loads. Likewise, the spine is not loaded by individual forces or moments but by combined dynamic loads (flexion, compression, torsion, and shear forces) in different spatial directions.

The different loads exerted on the individual segments of the spine are reflected by fracture rates. Fractures were mainly observed in the thoracic spine (TV 7 and TV 8) and the zone of transition between the thoracic and lumbar spine (TV 12 to LV 1) (see Figure 3b). One explanation for this fracture cascade along the spine could be the predominant curvatures of the spine [28]. The turning point of curvature in thoracic kyphosis is located in the central portion of the thoracic spine (TV 7 and TV 8). A more pronounced curvature is associated with higher bending moments and compression forces. This notion is supported by the fact that an increase in the radius of the curvature results in greater bending loads; this phenomenon leads to greater kyphosis that develops over the course of one’s life and is especially significant in osteoporosis. Likewise, the higher risk of fractures in zone TV 12 to LV 1 can possibly be explained by the greater mobility of the lumbar spine, leading to a higher compression load.

The clinical relevance of the data reported here should be examined in future studies. Current research on the trabecular bone score (TBS) is interesting. This score is a gray-scale parameter of bone texture using a modified experimental variogram that can be extracted from dual-energy X-ray absorptiometry (DXA) images [29]. The TBS was significantly correlated with BVF and SMI but not with bone mineral content or BMD. This score was also significantly correlated with rigidity, independent of bone mass. In multiple regression models, the combination of TBS, Tb.Th, and bone mineral content explained 79% of variability in rigidity. This was markedly superior to any other individual parameter [29].

One of the purposes of the present investigation was to determine whether different puncture directions in a comparable group of people of advanced age would yield similar data on microarchitecture. Similar data were found only for BVF, Tb.Sp., Conn.D, and SMI. In contrast, differences were seen in BVF, Tb.Th, and DA. The VD puncture may have yielded more subcortical structures, which were especially pronounced in the marginal areas of the vertebra. Furthermore, the Bland–Altman plot showed no incidental variance in data around the mean value. Rather, there was a funnel-shaped bias of data, revealing markedly different values at a higher BVF (see Figure 5). This factor should be considered in future investigations.

## 5. Conclusions

- Due to the unique and unchanging microstructure of cervical vertebrae with age, fractures in this region are extremely rare, even in the presence of osteoporosis.
- The different puncture directions used when obtaining cancellous bone from the vertebrae revealed an inhomogeneous microarchitecture inside the vertebrae.
- The highest bone volume fraction was seen in the marginal subcortical areas. Moreover, there were differences when using different puncture directions.
- For clinical practice using QCT measurements, at a cancellous bone mineral density of <60 mg/cm^3^ in the LS, we observed frequent insufficiency fractures in the spine, with the exception of the CS.
- The differences in bone volume fraction between the center of the vertebra and its marginal regions, as well as between the individual segments of the spine, helped explain fractures in the fish vertebrae.

### Limitations

The present study was a comparative descriptive investigation on an existing body of material using a fixed number of cases. Consequently, complex statistical procedures could not be used extensively. Only body donors of advanced age were investigated. Therefore, the results of this study permit no statements to be made about the bone structure of younger body donors. We also had very little data on the medical histories of the donors, especially the administered type and duration of the drugs or physical treatment used to treat osteoporosis.

## Figures and Tables

**Table 1:** Medical history.

	Overall Group (*n* = 20)	CC Group (*n* = 10)	VD Group (*n* = 10)	*p*-Value
Age (years)	79.4 ± 6.4	79.0 ± 5.4	79.8 ± 7.6	0.788 ^T^
Gender (male/female)	6/14	2/8	4/6	0.329 ^C^
Body mass index (kg/m^2^)	24.9 ± 6.6	27.3 ± 7.0	22.4 ± 5.5	0.099 ^T^
Bone mineral density in the LS (mg/cm^3^) *	53.2 (44.3–64.2)	57.5 (43.6–69.6)	51.7 (45.3–64.1)	0.971 ^M^
Median number of fractures	2.0 (1.0—2.0)	1.5 (0.75–2.0)	2.0 (0.75–3.0)	0.393 ^M^
Excluded segments	C5, C6, T8, T12, L1, L3	C5, C6, T8, T12, L1, L3	C5, C6, T8, T12, L1, L3	
Number of investigated vertebrae (*n*)	120	60	60	
Number of investigated cancellous bone cylinders (*n*)	360	180	180	

Results are shown for non-normally distributed parameters as median with the 1st and 3rd quartiles (Q1–Q3), and for normally distributed parameters as mean ± standard deviation (M ± SD). CC, craniocaudal; VD, ventrodorsal; C, cervical; T, thoracic; L, lumbar; * Quantitative computed tomography (QCT) measurements; T, independent Student’s *t*-test; C, chi-squared test; M, Wilcoxon-Mann-Whitney test.

**Table 2:** Descriptive statistics of micro-CT parameters of intervertebral quadrant comparisons for the individual segments with reference to the direction of puncture.

Quadrants						Group Comparison		
Parameter	Spinal Segments	Overall M ± SD	Q I M ± SD	Q II M ± SD	Q III M ± SD	Q I vs. Q II *p*-Value	Q III vs. Q II *p*-Value	Q I vs. Q III *p*-Value
Parametric variables								
BVF (%)	Overall CC VD	17.07 ± 4.69	18.49 ± 4.80	16.99 ± 4.74	15.72 ± 4.97			
		20.39 ± 3.31	22.41 ± 2.19	17.91 ± 2.92	20.86 ± 3.38	0.016 *	0.095	0.364
	CS CC VD	22.73 ± 2.46	23.65 ± 4.87	22.83 ± 0.36	21.72 ± 1.63			
		21.82 ± 2.74	21.99 ± 3.29	20.55 ± 2.02	22.91 ± 4.10			
	TS CC VD	13.40 ± 2.10	14.94 ± 2.61	12.84 ± 0.90	12.41 ± 2.64			
		20.98 ± 3.63	24.24 ± 1.35	18.01 ± 2.63	20.70 ± 4.24			
	LS CC VD	15.07 ± 2.21	16.88 ± 0.10	15.31 ± 1.75	13.03 ± 2.33			
		18.38 ± 2.98	21.0 ± 0.52	15.18 ± 1.62	18.97 ± 2.55			
Tb.Th (µm)	Overall CC	159 ± 16	158 ± 15	169 ± 19	151 ± 10			
	VD	179 ± 14	186 ± 12	169 ± 9	181 ± 14			
	CS CC	173 ± 19	169 ± 21	193 ± 3	159 ± 14			
	VD	177 ± 12	181 ± 17	168 ± 7	182 ± 13			
	TS CC	148 ± 9	146 ± 12	151 ± 8	146 ± 11			
	VD	185 ± 10	195 ± 8	178 ± 6	183 ± 13			
	LS CC	158 ± 8	159 ± 2	165 ± 2	148 ± 3			
	VD	174 ± 17	184 ± 12	160 ± 3	177 ± 25			
Tb.Sp (µm)	Overall CC VD	549 ± 40	522 ± 24	564 ± 39	561 ± 43			
		557 ± 38	553 ± 46	548 ± 45	570 ± 21			
	CS CC VD	524 ± 21	519 ± 11	534 ± 37	519 ± 22			
		564 ± 44	591 ± 48	521 ± 27	581 ± 29			
	TS CC VD	576 ± 41	540 ± 35	589 ± 23	599 ± 49			
		552 ± 46	505 ± 2	587 ± 52	563 ± 33			
	LS CC VD	547 ± 40	506 ± 16	568 ± 52	566 ± 2			
		555 ± 28	562 ± 25	536 ± 45	567 ± 8			
Conn.D (mm^−3^)	Overall CC	6.03 ± 2.30	6.95 ± 2.52	5.65 ± 1.49	5.45 ± 2.80			
	VD	5.92 ± 1,58	5.93 ± 0.10	5.95 ± 1.76	5.89 ± 1.75			
	CS CC	8.44 ± 1.64	9.35 ± 2.69	7.11 ± 0.74	8.86 ± 0.25			
	VD	6.39 ± 1.31	5.57 ± 0.86	7.20 ± 1.78	6.41 ± 1.42			
	TS CC	4.86 ± 1.80	6.33 ± 0.66	4.92 ± 2.12	3.32 ± 1.46			
	VD	6.39 ± 2.27	6.97 ± 2.77	5.88 ± 2.52	6.33 ± 3.24			
	LS CC	4.79 ± 1.24	5.15 ± 2.28	5.04 ± 0.02	4.18 ± 1.15			
	VD	4.99 ± 0.27	5.26 ± 0.19	4.76 ± 0.03	4.94 ± 0.30			
DA (n)	Overall CC VD	1.87 ± 0.20	1.89 ± 0.21	1.86 ± 0.21	1.84 ± 0.23			
		1.20 ± 0.62	1.69 ± 0.10	0.36 ± 0.03	1.60 ± 0.11	<0.001 ***	<0.001 ***	0.379
	CS CC VD	1.66 ± 0.09	1.74 ± 0.12	1.66 ± 0.10	1.60 ± 0.02			
		1.16 ± 0.63	1.64 ± 0.16	0.34 ± 0.06	1.49 ± 0.05	<0.001 ***	<0.001 ***	0.047
	TS CC VD	2.01 ± 0.15	1.84 ± 0.08	2.11 ± 0.05	2.08 ± 0.13			
		1.22 ± 0.65	1.60 ± 0.07	0.38 ± 0.03	1.67 ± 0.14	0.001**	0.001 **	0.526
	LS CC VD	1.92 ± 0.18	2.09 ± 0,26	1.83 ± 0.04	1.85 ± 0.12			
		1.24 ± 0.69	1.71 ± 0.20	0.36 ± 0.01	1.65 ± 0.01	0.001 **	0.001 **	0.637
SMI	Overall CC VD	1.87 ± 0.28	1.78 ± 0.31	1.95 ± 0.28	1.89 ± 0.27			
		1.83 ± 0.19	1.78 ± 0.17	1.92 ± 0.19	1.79 ± 0.21			
	CS CC VD	1.52 ± 0.13	1.40 ± 0.20	1.59 ± 0.01	1.56 ± 0.08			
		1.64 ± 0.13	1.57 ± 0.01	1.79 ± 0.04	1.54 ± 0.10			
	TS CC VD	2.05 ± 0.08	1.95 ± 0.02	2.09 ± 0.07	2.10 ± 0.01			
		1.87 ± 0.13	1.88 ± 0.09	1.83 ± 0.20	1.89 ± 0.17			
	LS CC VD	2.06 ± 0.10	2.0 ± 0.02	2.16 ± 0.12	2.01 ± 0.08			
		1.99 ± 0.12	1.90 ± 0.03	2.14 ± 0.06	1.94 ± 0.01			

The data refer to normally distributed parameters as mean values ± standard deviation (M ± SD); CS, cervical spine; TS, thoracic spine; LS, lumbar spine; BVF, bone volume fraction; Tb.Th, trabecular thickness; Tb.Sp, trabecular separation; Conn.D, connectivity density; DA, degree of anisotropy; SMI, structure model index; CC, craniocaudal puncture; VD, ventrodorsal puncture; Q I, Quadrant I; Q II, Quadrant II; Q III, Quadrant III; P post-hoc LSD test. The levels of significance are *p* < 0.05 *, *p* < 0.01 ** and *p* < 0.001 ***.

**Table 3:** Descriptive statistics of micro-CT parameters on longitudinal comparison of spinal segments.

	Spinal Segments				Group Comparison		
Parameter	Overall M ± SD	CS M ± SD	TS M ± SD	LS M ± SD	CS vs. TS *p*-Value	CS vs. LS *p*-Value	TS vs. LS *p*-Value
BVF (%) CC	17.07 ± 4.69	22.73 ± 2.46	13.40 ± 2.10	15.07 ± 2.21	<0.001 ***	<0.001 ***	0.218
VD	20.39 ± 3.31	21.82 ± 2.74	20.98 ± 3.63	18.38 ± 2.98			
Tb.Th (µm) CC	159 ± 16	173 ± 19	148 ± 9	158 ± 8	0.003 **	0.049 *	0.201
VD	179 ± 14	177 ± 12	185 ± 10	174 ± 17			
Tb.Sp (µm) CC	549 ± 40	524 ± 21	576 ± 41	547 ± 40			
VD	557 ± 38	564 ± 44	552 ± 46	555 ± 28			
Conn.D (mm^−3^) CC	6.03 ± 2.30	8.44 ± 1.64	4.86 ± 1.80	4.79 ± 1.24	0.001 **	0.001 **	0.943
VD	5.92 ± 1.58	6.39 ± 1.31	6.39 ± 2.27	4.99 ± 0.27			
DA (n) CC	1.87 ± 0.20	1.66 ± 0.09	2.01 ± 0.15	1.92 ± 0.18	0.001 **	0.007 **	0.305
VD	1.20 ± 0.62	1.16 ± 0.63	1.22 ± 0.65	1.24 ± 0.69			
SMI CC	1.87 ± 0.28	1.52 ± 0.13	2.05 ± 0.08	2.06 ± 0.10	<0.001 ***	<0.001 ***	0.871
VD	1.83 ± 0.19	1.64 ± 0.13	1.87 ± 0.13	1.99 ± 0.12	0.007 **	<0.001 ***	0.110

The data refer to normally distributed parameters as mean values ± standard deviation (M ± SD); CS, cervical spine; TS, thoracic spine; LS, lumbar spine; BVF, bone volume fraction; Tb.Th, trabecular thickness; Tb.Sp, trabecular separation; Conn.D, connectivity density; DA, degree of anisotropy; SMI, structure model index; CC, craniocaudal puncture; VD, ventrodorsal puncture; Q I, Quadrant I; Q II, Quadrant II; Q III, Quadrant III; P post-hoc LSD test. The levels of significance are *p* < 0.05 *, *p* < 0.01 ** and *p* < 0.001 ***.

**Table 4:** Group comparison of the micro-CT parameters of the spine according to craniocaudal and ventrodorsal puncture.

	Group CC (n = 10)	Group VD (n = 10)	*p*-Value
BVF (%)	17.07 ± 4.69	20.39 ± 3.31	0.018 *
Tb.Th (µm)	159 ± 16	179 ± 14	<0.001 ***
Tb.Sp (µm)	549 ± 40	557 ± 38	0.541
Conn.D (mm^−3^)	6.03 ± 2.30	5.92 ± 1.58	0.874
DA (*n*)	1.87 ± 0.20	1.20 ± 0.62	<0.001 ***
SMI	1.87 ± 0.28	1.83 ± 0.19	0.599

The data apply for normally distributed parameters as mean values ± standard deviation (M ± SD); BVF, bone volume fraction; Tb.Th, trabecular thickness; Tb.Sp, trabecular separation; Conn.D, connectivity density; DA, degree of anisotropy; SMI, structure model index; CC, craniocaudale puncture; VD, ventrodorsale puncture; P, independent Student’s *t*-test. The levels of significance are *p* < 0.05 * and *p* < 0.001 ***.

## Data Availability

The data presented in this study are available upon request from the corresponding author.

## Abbreviations

Al	Aluminum
BMD	Bone mineral density
BMI	Body mass index
BVF	Bone volume fraction
cm	Centimeter
CC	craniocaudal
CS	Cervical spine
CT	Computed tomography
CV	Cervical vertebra
Conn.D	Connectivity density
DA	Degree of anisotropy
DXA	Dual-energy X-ray absorptiometry
Fig.	Figure
Fs	Fracture
g/cm^3^	Gram/cubic centimeter
kV	Kilovolt
LV	Lumbar vertebra
LS	Lumbar spine
mg/cm^3^	Milligram/cubic centimeter
mg/mL	Milligram/milliliter
Micro-CT	Micro-computed tomography
Mio.	Million
mL	Milliliter
mm	Millimeter
M	Mean value
OP	Osteoporosis
Q I	Quadrant I
Q II	Quadrant II
Q III	Quadrant III
QCT	Quantitative computed tomography
ROI	Region of interest
SD	Standard deviation
SMI	Structure model index
TS	Thoracic spine
TV	Thoracic vertebra
Tb.N	Trabecular number
Tb.Pf	Trabecular pattern factor
Tb.Sp	Trabecular separation
Tb.Th	Trabecular thickness
VD	Ventrodorsal
VFs	Vertebral fractures
µm	Micrometer
µA	Microampere
